# Intracavitary brachytherapy with additional Heyman capsules in the treatment of cervical cancer

**DOI:** 10.1007/s00404-022-06602-4

**Published:** 2022-05-31

**Authors:** Sophia Scharl, Christine Hugo, Clara-Bianca Weidenbächer, Holger Bronger, Christine Brambs, Marion Kiechle, Marcus R. Makowski, Stephanie E. Combs, Lars Schüttrumpf

**Affiliations:** 1 Department of Radiation Oncology, Technische Universität München (TUM), Ismaninger Straße 22, Munich, Germany; 2 Department of Radiation Oncology, University Hospital Ulm, Albert-Einstein-Allee 23, Ulm, Germany; 3 Department of Obstetrics and Gynecology, Technische Universität München (TUM), Ismaninger Straße 22, Munich, Germany; 4 Department of Diagnostic and Interventional Radiology, Klinikum rechts der Isar, Technische Universität München, Munich, Germany; 5 Deutsches Konsortium für Translationale Krebsforschung (DKTK), Partner Site Munich, Munich, Germany; 6 Institute of Radiation Medicine (IRM), Helmholtz Zentrum München, Ingolstädter Landstraße 1, Neuherberg, Germany

**Keywords:** Cervical cancer, Brachytherapy, Heyman capsules, Tandem-ring applicator

## Abstract

**Purpose:**

Brachytherapy is a mandatory component of primary radiochemotherapy in cervical cancer. The dose can be applied with a traditional intracavitary approach (IC alone) or with multiple catheter brachytherapy to optimize dose distribution in an individual concept. We therefore evaluated whether the utilization of a tandem–ring applicator plus additional intracavitary applicators (add IC) provides an advantage over the traditional IC alone approach, as this method is less time consuming and less invasive compared to a combined intracavitary/interstitial brachytherapy.

**Methods:**

Twenty three procedures of intracavitary brachytherapy for cervical cancer with additional intracavitary applicators performed in seven patients treated between 2016 and 2018 in our institution were included in this study. Plans were optimized for D90 HR-CTV with and without the utilization of the additional applicators and compared by statistical analysis.

**Results:**

D90 for HR-CTV was 5.71 Gy (±1.17 Gy) for fractions optimized with add IC approach and 5.29 Gy (±1.24 Gy) for fractions without additional applicators (*p* < 0.01). This translates to a calculated mean EQD2 HR-CTV D90 of 80.72 Gy (±8.34 Gy) compared to 77.84 Gy (±8.49 Gy) after external beam therapy and four fractions of brachytherapy for add IC and IC alone, respectively (*p* < 0.01). The predictive value of improved coverage of HR-CTV in the first fraction was high.

**Conclusion:**

In a subgroup of cases, the addition of intracavitary Heyman capsules can be an alternative to interstitial brachytherapy to improve the plan quality compared to standard IC alone brachytherapy. The benefit from the addition of applicators in the first fraction is predictive for the following fractions.

**Supplementary Information:**

The online version contains supplementary material available at 10.1007/s00404-022-06602-4.

## Introduction

Intracavitary brachytherapy is a mandatory component of primary radiochemotherapy in cervical cancer as it improves local control and subsequently overall survival [1–3]. Although brachytherapy is one of the oldest techniques in radiation oncology, the benefit is undoubtable and no other modern radiation technique can offer the same precision of local high-dose deposition and sparing of normal tissue. The technical improvements of percutaneous irradiation in intensity modulation (IMRT) and the more precise application by imaging (IGRT) nowadays allow better protection of organs at risk with smaller setup margins, but cannot compensate for the physical advantages of modern brachytherapy. In terms of overall survival, a hypofractionated percutaneous dose application (stereotactic body radiotherapy) appears to be equivalent to brachytherapy, but results in high unacceptable toxicity rates [3, 4]. In recent years, numerous advances have been implemented in brachytherapy as well. Image-guided brachytherapy has enabled physicians to optimize their target volume and precisely locate the organs at risk resulting in improvement of local control and reduced side effects compared to the traditional prescription to a two-dimensional geometric point (point A dosimetry) [5]. Contouring guidelines have been published by the GEC-ESTRO to standardize target volume definition [6, 7].

For cervix cancer brachytherapy, a variety of applicators exist. The most common ones for intracavitary brachytherapy are the tandem/ovoid and tandem/ring applicators. Applicator choice depends the anatomy at the introitus, exocervix and vaginal fornices, but also on the availability of a certain system at the institution and physicians’ preferences. The advantage of tandem/ovoid treatment lies in the availability of different sizes, thus allowing for configuration adjustments, whereas the tandem-ring applicator offers a higher reproducibility and can be of advantage in patients with an anteroposterior tumor spread in the vaginal fornix due to the option of positioning the source over an angle of 360°. However, it has become clear that in patients with large primary tumors or unfavorable anatomy, the desired target volume coverage cannot be achieved by any form of intracavitary applicator [3, 8]. To overcome the issue of inadequate dose coverage, combined intracavitary and interstitial brachytherapy applicators such as the tandem–ring Vienna applicator and the tandem-ovoid Utrecht applicator have been developed that include the option of needle placement into the parametria [9, 10]. In a further development, the Venezia applicator (Elekta, Sweden) was introduced, offering a template within the ring for the guidance of parallel and/or oblique interstitial needles for even more advanced tailoring of dose distribution [11]. Other centers apply freehand insertion of metal needles [3, 8]. Interstitial brachytherapy consistently improved plan quality leading to higher target volume doses and better coverage while sparing surrounding organs at risk. While multiple catheter brachytherapy is being used by many centers, others refrain from applying brachytherapy at all as it requires expensive equipment and trained physicians [2]. In general, accurate implantation of interstitial needles is difficult and time-consuming [8]. Subsequently, a large number of centers are currently not applying interstitial brachytherapy, although brachytherapy is considered mandatory based on all cervical cancer treatment guidelines [1, 6, 12, 13]. Therefore, we intended to evaluate whether the utilization of additional intracavitary applicators (add IC) provides an advantage over the traditional tandem–ring applicator (IC alone), as it is a method that requires little additional time and experience compared to standard intracavitary brachytherapy. Therefore, it can be performed at any center carrying out intracavitary brachytherapy at the moment.

## Material and methods

### Patients

Twenty three (23) procedures of intracavitary brachytherapy for cervical cancer with additional intracavitary applicators (add IC) performed in seven consecutive patients treated between 2016 and 2018 in our institution were included in this study. In six patients with 21 procedures, two applicators were used, and in one patient with two procedures only one additional applicator was used. Patients’ characteristics are shown in Table 1. Written informed consent in the use of scientific data was obtained by all patients. This study was approved by the Ethics Committee of the Technical University of Munich.

**Table 1:** Patients’ characteristics

Mean age (years)	53 ± 5.2
Histology	
Squamous cell cancer	7 (100%)
Grading	
G1	0
G2	3 (43%)
G3	4 (57%)
Tumor stage	
*T*	
T1b2	1 (14%)
T2a	1 (14%)
T2b	4 (57%)
T3	0
T4	1 (14%)
*N*	
N0	2 (29%)
N1	5 (71%)
*M*	
M0	7 (100%)
Size of primary tumor pretreatment MRI (cm)	
Craniocaudal	6.8 ± 3.0
Anteroposterior	5.7 ± 0.8
Lateral	6.4 ± 1.5
Size of primary tumor planning MRI (cm)	
Craniocaudal	2.0 ± 2.1
Anteroposterior	2.2 ± 1.3
Lateral	3.3 ± 1.4

### Brachytherapy applicators

Brachytherapy was performed under general anesthetic by a gynecological oncologist and radiation oncologist. The cervical canal was dilated by Hegar pens (MEDICON eG, Germany). One to two Heyman capsules (Varian Medical Systems, Palo Alto, CA, USA) were inserted into the uterus prior to the tandem and ring. A traditional tandem–ring applicator (Titanium Vienna-style ring applicator, Varian Medical Systems, Palo Alto, CA, USA) was used. Three angles (30°, 45° and 60°) for ring and tandem and three different lengths (2 cm, 4 cm, 6 cm for the tandem) were available. The applicator was chosen by the treating physician depending on patients’ anatomy.

### Imaging and planning procedures

**Fig. 1:**
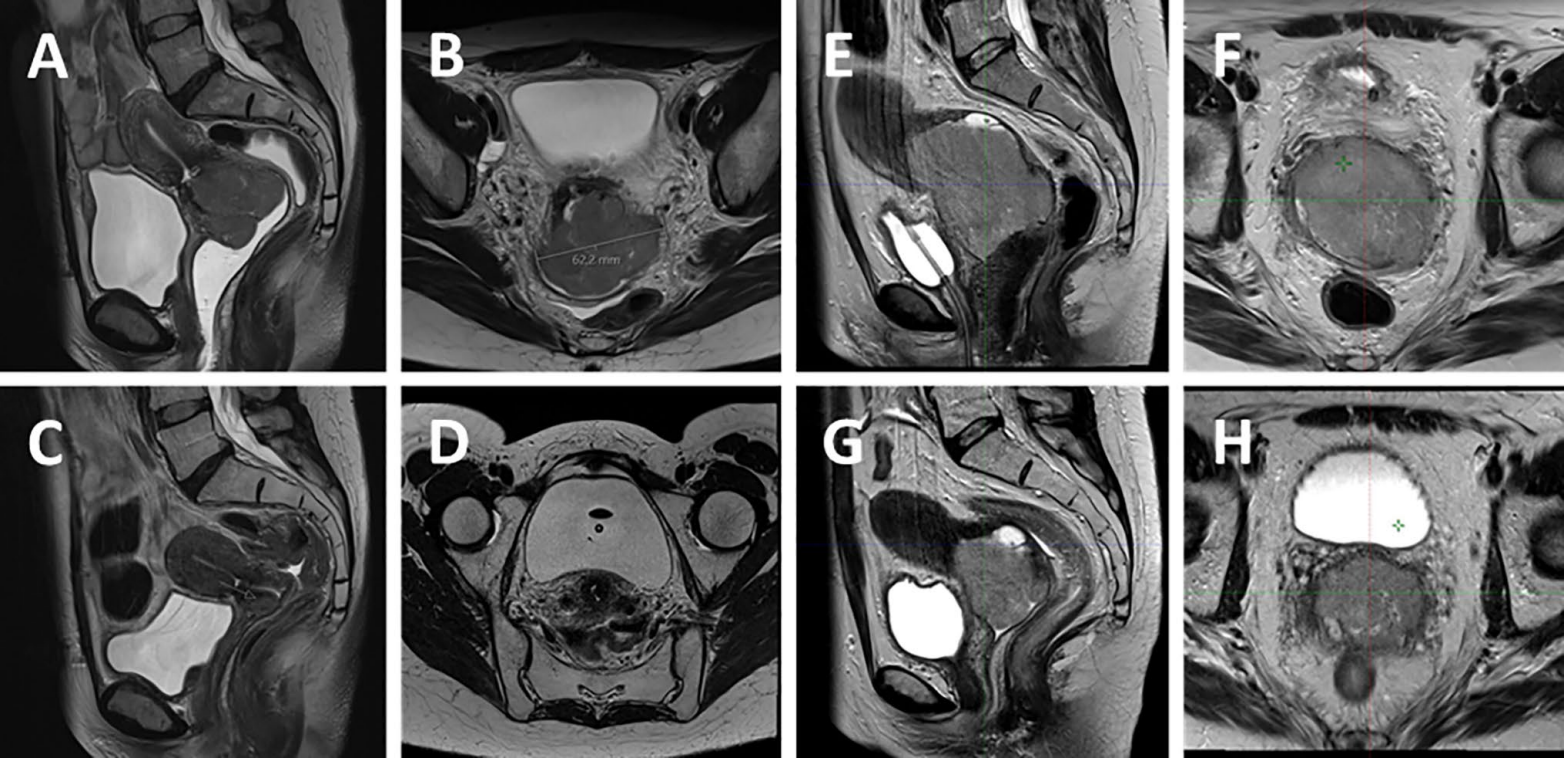
An example of a fast-responding tumor before starting percutaneous radiotherapy (**A** and **B**) and after 40 Gy was applied (**C** and **D**). An example of a slow-responding tumor before starting percutaneous radiotherapy (**E** and **F**) and after 40 Gy was applied (**G** and **H**)

A pretreatment magnetic resonance imaging (MRI) or positron emission tomography (PET)–MRI was obtained not more than 1 week before the start of radiochemotherapy. At approximately 40–45 Gy prior to the first brachytherapy procedure, another MRI was performed (Fig. 1). MRIs and PET–MRIs were obtained at the radiology department or department of nuclear medicine of our institution.

**Fig. 2:**
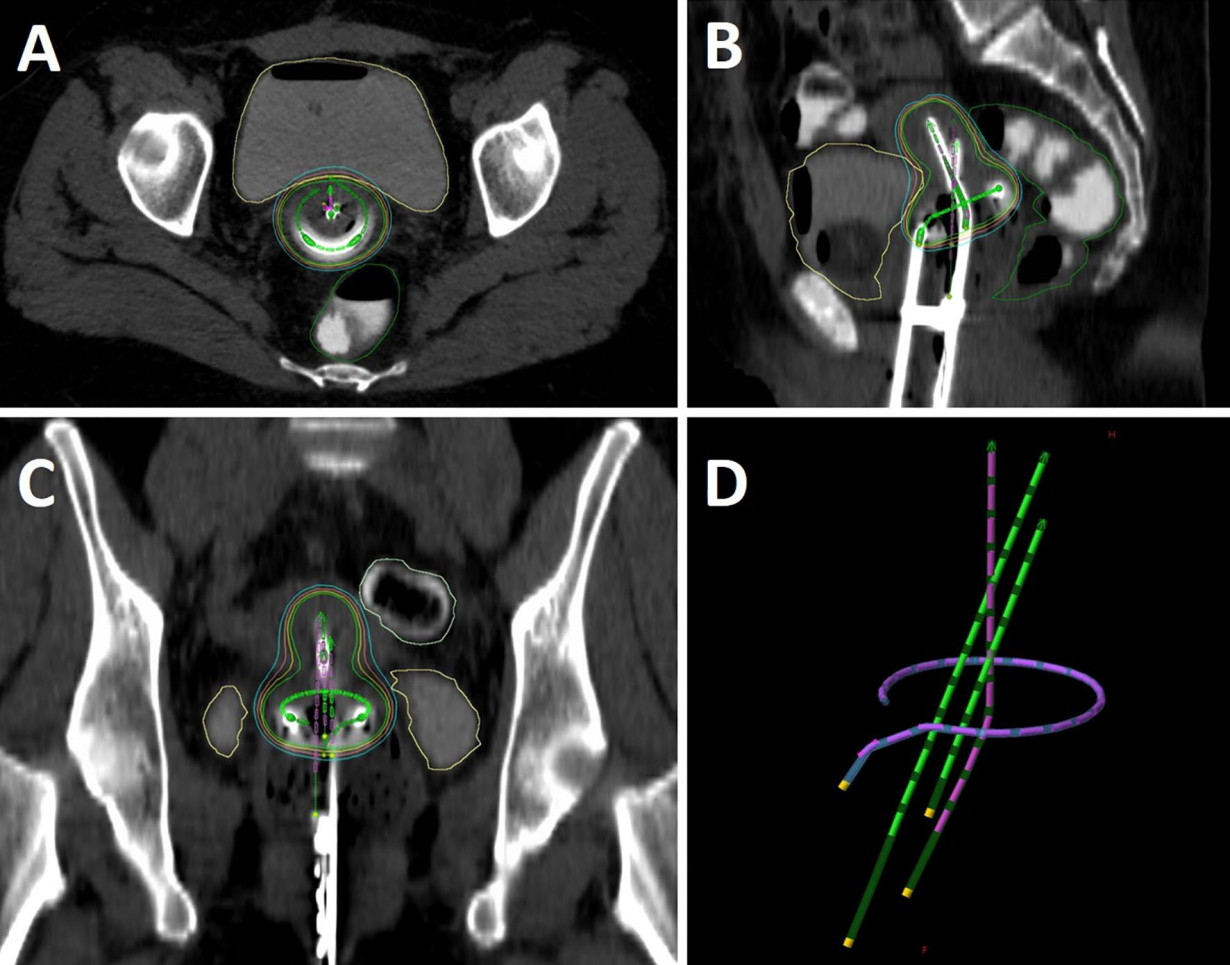
Axial (**A**), sagittal (**B**) and coronal (**C**) views of planning CT with tandem–ring applicator and two additional intracavitary applicators. Three-dimensional reconstruction of the tandem–ring applicator (purple) and additional applicators (green) (**D**)

A treatment planning computed tomography (CT) was carried out on the day of brachytherapy with brachytherapy applicators in position using a Somatom Emotion CT-scanner (Siemens, Germany) (Fig. 2). To facilitate contouring of organs at risk, the bladder and rectum were filled with 5 ml of contrast medium (Telebrix Gastro, Guerbet, Germany) diluted in 115 ml and 45 ml of sterilized water, respectively. The additional applicators were labeled with numbers and equipped with distinct radiopaque wires.

Pretreatment and pre-brachytherapy MRI were coregistered to the planning CT. High-risk clinical target volume (HR-CTV), intermediate-risk clinical target volume (IR-CTV), residual gross tumor volume (GTVres) and the organs at risk (OAR) were contoured according to GEC-ESTRO Guidelines [6, 7]. Treatment planning was carried out using Eclipse treatment planning software (version 13.0.33; Varian Medical Systems, Palo Alto, CA, USA). For each single brachytherapy fraction, two different plans were generated utilizing the tandem–ring applicator and the additional intracavitary applicators (add IC) or the tandem–-ring applicator alone (IC alone). Planning aims and constraints are described in Table 2. Plans were optimized to reach the indicated dose coverage of HR-CTV D90 without exceeding the tolerance doses of organs at risk. If the intended dose of HR-CTV was reached, the plan was further optimized to improve D98 GTVres and D98 HR-CTV (in this order).

**Table 2:** Planning aims and constraints

	EQ D2	Dose per fraction
Planning aims		
D90 HR-CTV	> 85 Gy	6.5 Gy
D 98 GTVres	> 90 Gy	7.1 Gy
D98 HR-CTV	> 75 Gy	5.1 Gy
Constraints (D2 cm^3^)		
Bladder	< 90 Gy	5.8 Gy
Rectum	< 75 Gy	4.4 Gy
Sigmoid	< 75 Gy	4.4 Gy
Bowel	< 70 Gy	3.9 Gy

The theoretical equivalent dose in two gray fractions (EQD2) for each brachytherapy fraction was calculated using the EQD2 model, with an *α*/*β* of 10 for tumor and *α*/*β* of 3 for normal tissue. A previous external beam therapy of 50.4 Gy in 28 fractions as well as the dose of the corresponding brachytherapy fraction multiplied by four were taken into account. This method of calculating EQD2 doses for each fraction separately was used, since not every patient received brachytherapy with additional Heyman capsules in every fraction. Nevertheless, information on EQD2 doses is essential, as it is the relevant quantity to estimate the benefit for local control [14].

The chosen dose constraints were based on the EMBRACE II study [15].

### Statistical evaluation

Continuous data were expressed as means ± standard deviation (SD). Comparisons were made by two-sided paired *t* test for dependent variables and unpaired *t* test was used for independent samples. A *p* value of 0.05 was defined as the threshold for statistical significance within a confidence interval of 95%. All calculations and figures were done with the software packages SPSS 23 (IBM, USA).

## Results

### HR-CTV

**Fig. 3:**
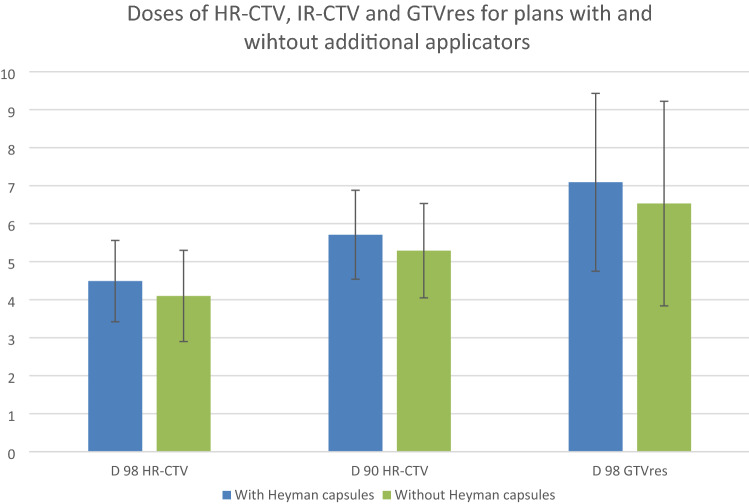
Diagrammatic presentation of the mean doses of HR-CTV and GTVres for plans with and wihtout additional applicators

D90 HR-CTV was 5.71 Gy (±1.17 Gy) for fractions optimized with add IC approach and 5.29 Gy (±1.24 Gy) for fractions with IC alone (*p* < 0.01). The mean improvement achieved by applying additional applicators was 0.42 Gy (±0.49 Gy). In seven fractions, an improvement of more than 0.5 Gy and in ten fractions an improvement of 0.2–0.5 Gy per fraction was achieved. In six fractions, no benefit through the addition of applicators was reached. D98 HR-CTV was 4.49 Gy (±1.07 Gy) for fractions with add IC approach and 4.10 Gy (±1.20 Gy) for fractions with IC alone. This translates into a mean of calculated EQD2 HR-CTV D90 of 80.72 Gy (±8.34 Gy) compared to 77.84 Gy (±8.49 Gy) with or without additional applicators, respectively (*p* < 0.01) (Fig. 3). Patients with a benefit of 0.5 Gy in the first fraction D90 HR-CTV was 77.65 Gy (±9.66 Gy) without compared to 83.18 Gy (±8.93 Gy) with the additional applicators.

V100% HR-CTV was 74.14% (±14.75%) and 70.98% (±14.72%) for patients with and without additional applicators, respectively (*p* < 0.01).

### GTVres

D98 and D90 GTVres were 7.09 Gy (±2.34 Gy) and 8.84 Gy (±2.59 Gy) for fractions with add IC and 6.53 Gy (±2.69 Gy) and 7.72 Gy (±2.86 Gy) for fractions with IC alone, respectively (*p* < 0.01). EQD2 for D98 GTVres was 92.53 Gy (±17.94 Gy) and 88.66 Gy (±19.15 Gy) for add IC and IC alone, respectively (*p* < 0.01). Patients with a benefit of 0.5 Gy in the first fraction D98 GTVres was 80.11 Gy (±17.97 Gy) without compared to 85.89 Gy (±13.94 Gy) with the additional applicators. Further mean values are described in Table 3.

**Table 3:** Doses of HR-CTV, IR-CTV and GTVres for plans with and wihtout additional applicators

	With Heyman capsules	Without Heyman capsules	*p*
*Target volume (Gy per fraction/V100 in percent of volume)*			
HR-CTV			
D100 HR-CTV	2.95 ± 0.64	2.79 ± 0.84	0.13
D98 HR-CTV	4.49 ± 1.07	4.10 ± 1.20	0.01*
D90 HR-CTV	5.71 ± 1.17	5.29 ± 1.24	< 0.01*
D50 HR-CTV	9.53 ± 1.79	8.91 ± 1.49	< 0.01*
V100 HR-CTV	74.14 ± 14.75	70.98 ± 14.72	< 0.01*
IR-CTV			
D100 IR-CTV	2.11 ± 0.84	2.09 ± 0.91	0.75
D98 IR-CTV	2.91 ± 1.10	2.82 ± 1.20	0.26
D90 IR-CTV	4.00 ± 1.16	3.92 ± 1.61	0.67
GTVres			
D 100 GTVres	5.82 ± 2.23	5.51 ± 2.47	0.04*
D 98 GTVres	7.09 ± 2.34	6.53 ± 2.69	< 0.01*
D 90 GTVres	8.48 ± 2.59	7.72 ± 2.86	< 0.01*
Target volume (EQD2)			
D 98 HR-CTV	72.44 ± 6.73	70.11 ± 7.14	0.01*
D 90 HR-CTV	80.72 ± 8.34	77.84 ± 8.49	< 0.01*
D 98 GTVres	92.53 ± 17.94	88.66 ± 19.15	< 0.01*

Significant differences are labeled by *

### Organs at risk

The organ at risk limiting dose escalation in the majority of cases was the bladder with mean doses of 5.50 Gy (±0.41 Gy) and 5.65 Gy (±0.35 Gy) for fractions with add IC and IC alone (*p* = 0.02). There were no significant differences in the mean doses of the remaining organs at risk (Table 4).

**Table 4:** Doses of organs at risk

	With Heyman capsules	Without Heyman capsules	*p*
Organs at risk (D2 cm^3^)			
Bladder	5.50 ± 0.41	5.65 ± 0.35	0.02*
Rectum	3.79 ± 0.66	3.72 ± 0.68	0.45
Sigmoid	2.87 ± 1.07	2.79 ± 1.08	0.06
Bowel	2.40 ± 1.05	2.32 ± 1.09	0.43
Organs at risk (D2 cm^3^)			
Bladder	7.22 ± 0.90	7.37 ± 0.80	0.36
Rectum	4.98 ± 1.18	5.10 ± 1.06	0.55
Sigmoid	3.88 ± 1.64	3.77 ± 1.59	0.25
Bowel	3.30 ± 1.72	3.34 ± 1.72	0.78

Significant differences are labeled by *

### Feasibility

The insertion of additional Heyman capsules did not require additional training.

### Fractions with improved coverage of D90 HR-CTV

The predictive value of an improved coverage of HR-CTV in the first fraction was high. For patients with an improvement of more than 0.5 Gy in the first fraction, the mean improvement in the remaining fractions was 0.63 Gy (±0.52 Gy), whereas it was 0.07 Gy (±0.16 Gy) for patients without any dosimetric improvement with additional applicators. The mean improvement for the subsequent fractions in patients with an improvement of 0.2–0.5 Gy in the first fraction was 0.30 Gy (±0.13 Gy).

The initial GTV in the three patients that showed a clear improvement through the addition of Heyman capsules was slightly larger, particularly in cranio-caudal extension compared to the other patients (7.13 cm ± 3.78 cm vs 5.58 cm ± 2.71 cm). The difference was not statistically significant (*p* = 0.58). When only one Heyman capsule was used (2 fractions in one patient), no improved dose coverage was observed.

## Discussion

To the best of our knowledge, this is the first study examining the effect of inserting additional Heyman capsules to the traditional tandem–ring applicator when conducting an intracavitary brachytherapy in patients with cervical cancer. We observed a mean improvement of 0.42 Gy per fraction leading to a calculated mean EQD2 of 80.72 Gy compared to 77.84 Gy for D90 HR-CTV and 92.53 Gy compared to 88.66 Gy for D98 GTVres. In three of the seven7 analyzed patients, the benefit from the addition of Heyman capsules seemed to be greater compared to the others, whereas in three patients no improvement was observed. At the same time, the insertion of additional Heyman capsules did not require additional training.

Multiple studies have demonstrated the necessity to perform brachytherapy in patients treated by primary radio-chemotherapy for cervical cancer [1, 2]. The relationship between the applied dose and local control as well as patient survival is well understood [15, 16]. Particularly in patients with large primary tumors, doses exceeding 85 Gy for HR-CTV are essential.

In our cohort, the estimated overall benefit in local control according to Tanderup et al. would be around 2% for FIGO stage II patients, but rises to approximately 5–8% for FIGO stages III and IV [14]. Taking into account only patients that benefited from the additional applicators, the estimated improvement in local control is 5–10% for stages II–IV. In other patients, no benefit through additional applicators was achieved. Compared to combined intracavitary/interstitial brachytherapy with reported cumulative EQD2 D90 HR-CTV of approximately 87–90 Gy and increases of 5–10 Gy, our approach shows a considerably smaller improvement [8, 11, 17, 18]. The implementation, however, is easier and viable in any department that regularly performs intracavitary brachytherapy with a tandem–ring applicator. In combined IC/IS brachytherapy, needles are inserted directly into the tumor and, if necessary, parametria posing the risk of uterine perforation (Table S1). The method utilized in our institution is an adapted version of the Heyman packing method inserting the additional applicators into the uterine cavity via the cervical canal. This method demonstrated excellent results in patient populations treated by primary radiotherapy for endometrial cancer due to comorbidities [19, 20]. In comparison to the technique applied for endometrial cancer, we used only two capsules in addition to the traditional tandem–ring applicator, as only the cervix and the initial tumor extension need to be covered by an adequate dose [19–21].

The characteristics of patients that potentially benefit from this concept are yet to be defined. Since the benefit was relatively consistent over fractions, certain primary tumor and their spatial situations to the organs at risk are likely to be predictors for an advantage from this concept. In fractions with only one Heyman capsule, no profit was observed. Therefore, we recommend the usage of two Heyman capsules. The planning time and the time needed to place the applicators, on the other hand, increases when additional applicators are used. Furthermore, the exact position of the Heyman capsules is difficult to control beforehand, as the tube of the Heyman capsules that are positioned into the cervix is flexible. Therefore, the potential advantages in dose coverage should be weighed against the increased expenditure. Since patients that did not benefit from the procedure in the first fraction did not seem to benefit in further fractions, it seems reasonable to test the addition of applicators in the first fraction and decide whether to continue thereafter. Subsequent slipping of the Heyman capsules is unlikely, as they are fixed together with the tandem–ring applicator by using a tamponade.

The limitations of this study are the small patient and fraction numbers. These impede conclusions on the characteristics of patients that potentially benefit from this procedure. Further studies are needed to confirm our results and to identify a patient cohort with the highest benefit from this procedure.

## Conclusion

The therapeutic potential of brachytherapy in the curative treatment of cervical carcinoma should be fully exploited. The addition of Heyman capsules to intracavitary brachytherapy can be an alternative for centers to interstitial brachytherapy, as it is less invasive and implementation is easier. The first practical applications of this method already give an idea of the potential overall success. The treatment of patients with cervical carcinoma should be carried out in a certified center where all treatment modalities are available.

## Supplementary Information

Below is the link to the electronic supplementary material.

Supplementary file1 (DOCX 14 kb)

## Data Availability

The datasets generated and/or analyzed during the current study are not publicly available due to privacy regulations in the ethics approval, but are available from the corresponding author on reasonable request.
