# Supplementary material for: Intracavitary brachytherapy with additional Heyman capsules in the treatment of cervical cancer
Source: Arch Gynecol Obstet. 2022 May 31;307(2):557–64. doi: 10.1007/s00404-022-06602-4 (PMC9918574; doi:10.1007/s00404-022-06602-4)
Supplement: Supplementary file 1 — Supplementary file1 (DOCX 14 kb) [file 404_2022_6602_MOESM1_ESM.docx]

**Supplementary material**

Table S1: Overview of the two standard brachytherapy procedures. Advantages of a method are labelled with +, disadvantages with -.

|  | IC brachytherapy | IC/IS brachytherapy |
| --- | --- | --- |
| Target dose and dose conformity | - | + |
| Risk of complications | + | - |
| Availability | + | - |
| Time consumption | + | - |
